# Corrosion Behavior of 20G and TP347H in Molten LiCl-NaCl-KCl Salt

**DOI:** 10.3390/nano14121026

**Published:** 2024-06-13

**Authors:** Shijing Xie, Min Lei, Jiawei Sun, Chongdou Yang, Wenbo Liu, Di Yun, Xiqiang Zhao, Jie Qiu

**Affiliations:** 1 School of Nuclear Science and Technology, Xi’an Jiaotong University, Xi’an 710049, China; xieshijing@xjtu.edu.cn (S.X.); 425364@stu.xjtu.edu.cn (C.Y.); qiu2021@xjtu.edu.cn (J.Q.); 2 State Key Laboratory of Multiphase Flow, Xi’an Jiaotong University, Xi’an 710049, China; 3 National Engineering Laboratory for Reducing Emissions from Coal Combustion, Shandong University, Jinan 250061, China

**Keywords:** molten chloride salt, corrosion, oxide, steel

## Abstract

The corrosion behavior of 20G and TP347H materials was investigated in molten LiCl-NaCl-KCl salt. The corrosion rates of these materials in molten chloride salt are high and are strongly affected by the alloying surface oxide formation. The 20G shows uniform surface corrosion with almost no protective oxide formation on the surface. In contrast, the austenitic steel TP347H exhibits better corrosion resistance in molten chloride salts due to its high Cr content. Owing to the highly corrosive nature of molten chloride salts, the Cl^−^ in molten salt could react with oxides and alloy, inducing intergranular corrosion of austenitic steel in molten chloride salt environments.

## 1. Introduction

High-temperature molten salt has been widely used as a heat transfer fluid (HTF) in molten salt reactors, solar heat collection and factory heat recovery systems due to its favorable heat transfer and storage properties [1,2,3,4]. Numerous studies have investigated the heat transfer properties of molten salts, such as molten fluoride salt (FLiBe) [5,6,7], nitrate salt (NaNO_3_-KNO_3_) [8,9], carbonate salt (LiKCO_3_) [10] and chloride salt (LiCl-NaCl-KCl) [11]. In contrast, molten chloride salts have better thermophysical properties (density, viscosity, thermal conductivity, etc.), high thermal stability (800 °C) and lower cost (much lower than fluoride) [11,12], molten chloride salts (LiCl-NaCl-KCl, etc.) are commonly employed as electrolytes for spent fuel reprocessing and for electrolytic purification to recover nuclear waste in the nuclear industry [12]. However, molten chloride salt exhibits strong corrosion properties [11,12]. The corrosion of containers and metal structural materials in molten chloride salt presents a significant challenge, restricting the widespread application of molten salts [11,12,13,14].

In many corrosive environments, a thin passive film formed on a metal’s surface that acts as a barrier to reduce the corrosion of materials. However, in molten chloride salt, due to the high electronegativity and high activity of Cl^−^, the Cl^−^ leads to the oxide film crack and porous, reducing its effective adhesion and protection properties [15,16,17]. Shankar et al. studied the corrosion of 316L stainless steel in molten LiCl-KCl and found that the main corrosion of steel is the selective dissolution of Cr [18]. It is generally recognized that the corrosion of the alloys in molten chloride salt is the selective dissolution and/or oxidation of the elements depending on the different experimental conditions. To our knowledge, most of the corrosion studies are focused on purified chloride salts. Due to the hygroscopicity of the salts, O_2_/H_2_O are the most common impurities and are often involved in most chloride salt environments [19]. Although the corrosion behavior of alloys in molten salt has been examined extensively, the corrosion properties of alloys in molten chloride salt open to air are not well understood at present.

TP347H austenitic stainless steel, because of its excellent corrosion resistance, good high-temperature strength and creep properties, is widely used in superheaters, power plant boilers and biomass boilers [20,21,22,23]. Compared with TP347H, 20G is a typical low Cr carbon steel and is widely used in heating the surface of boilers and oil and natural gas transmission pipes owing to its low cost, thermal formability and weldability [24,25,26]. TP347H and 20G represent typical austenitic stainless steel and carbon steel, respectively, which could be used in molten salt environments [21,24]. However, the related researches of TP347H and 20G mainly focus on stress cracking corrosion (SCC), high-temperature water vapor corrosion and high-temperature combustion gas (CO_2_, HCl, SO_2_) corrosion [27,28,29,30,31]. The corrosion behavior of TP347H and 20G alloys in molten chloride salt environments open to air are scarcely studied and are therefore highly desirable.

In this paper, the high-temperature corrosion of 20G and TP347H in LiCl-NaCl-KCl ternary molten salts was systematically studied. The corrosion morphologies, corrosion products, phase composition, element distribution and other structures of the corroded samples were characterized and analyzed. The corrosion behavior of 20G and TP347H is compared and discussed.

## 2. Materials and Methods

### 2.1. Materials

The 20G and TP347H alloys used in this study were purchased from GoodFellow, and the alloy compositions are shown in Table 1. The specimens of 20G and HP347H alloys for the corrosion test were cut to samples with a dimension of 15 mm × 10 mm × 2 mm by electrospark wire-electrode cutting. Prior to the corrosion experiments, the specimens were ground using 600, 800, 1000 and 2000 grit emery paper in turn, followed by washing and cleaning with Etoh. After being dried, the sample dimensions were measured with an electronic caliber and weighed with an analytical balance with an accuracy of 0.01 mg.

### 2.2. Corrosion Experiment

LiCl (99.9% purity), NaCl (99.9% purity) and KCl (99.9% purity) powders were purchased from Aladdin to prepare the ternary eutectic salt with a melting point of 348 °C, consisting of 53.5 mol% LiCl, 8.6 mol% NaCland 37.9 mol% KCl [32,33]. For the corrosion experiment, 50 g of the eutectic chloride salt was prepared by mixing LiCl, NaCl and KCl in the above proper proportions, then loaded into an alumina crucible. Subsequently, we added the samples into the alumina crucible, transferred the crucible to a furnace and heated it to 700 °C for 50 h. After a 50 h corrosion experiment, we turned off the furnace and retrieved the specimens.

### 2.3. Analysis Method

After the corrosion experiment, the cross-sectional morphologies of specimens were observed using an FEI Quanta 3D FEG scanning electron microscope (SEM). The elemental distributions of the samples were characterized using Energy Dispersive Spectroscopy (EDS). The chemical compositions and structures of the samples after the experiment were characterized using a field emission FEI Talos F200X transmission electron microscopy (TEM). Note that the cross-sectional specimens for metallographic examination were prepared using standard grinding and polishing procedures to avoid damaging the corrosion products on the surface of the specimens during the sample preparation. The specimen was placed in a vacuum-free clear cold setting mold for cold setting solidification, ground using silicon carbide papers down to 2000 grit and then polished with 0.05 μm alumina slurry, and finally ultrasonically cleaned in ethanol and de-ionized water. TEM thin foils were cut using a FEI Quanta 3D focused ion beam (FIB).

## 3. Results

### 3.1. Corrosion Performance of Alloy Specimens in Molten Salt

**Figure 1:**
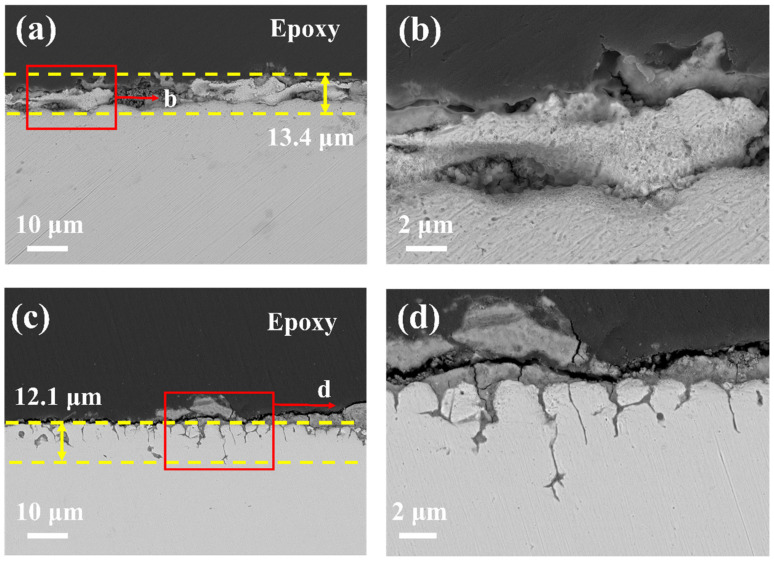
The cross-sectional morphologies of (**a**,**b**) 20G alloys and (**c**,**d**) TP347H alloys after exposure to molten LiCl-NaCl-KCl at 700 °C for 50 h. Note that the yellow dashed lines represent corrosion areas, and (**b**) and (**d**) correspond to zoom-in of the red rectangles in (**a**) and (**b**) respectively.

The SEM cross-sectional images of 20G and TP347H alloys after being exposed to molten chloride salt at 700 °C for 50 h are shown in Figure 1. Both 20G and TP347H alloys exhibit notable corrosion in the eutectic LiCl-NaCl-KCl melt at 700 °C. As shown in Figure 1a, the corrosion of 20G is general corrosion and the outer corrosion layer of 20G steel is severe, with a maximum corrosion depth of approximately 13.4 µm. Figure 1b reveals the presence of some internal corrosion pits, which first form near the outer wall of the steel and then propagate inward, further penetrating into the substrate. This is due to the dissolution of alloys in the near-surface area and providing pathways for the dissolved oxygen and electrolyte ingress into the substrate. The dissolved oxygen reacts with the substrate near the surface to form new corrosion products, continually deteriorating the steel substrate and inducing general corrosion.

In contrast, TP347H exhibits superior corrosion resistance in molten chloride salts. As illustrated in Table 1, the TP347H alloy contains higher levels of chromium (Cr) and nickel (Ni) compared to the 20G alloy. Previous studies have shown that the oxidation of Cr in the TP347H alloy could form spinel-structure oxides, which exhibit excellent corrosion resistance in corrosion environments [21]. Different from most corrosive environments, TP347H stainless steel does not form a stable oxide layer on its surface in molten chloride salt, as shown in Figure 1c. The corrosion of TP347H in molten chloride salt is mainly intergranular corrosion and the corrosion depth is approximately 12.1 µm. Similarly, Shi et al. [34] demonstrated that austenitic stainless steel has similar corrosion along grain boundaries (GBs) in molten chloride salts, indicating that austenitic stainless steel cannot form stable oxides in chloride molten salts, with its corrosion mainly activated dissolution corrosion of the alloy.

**Figure 2:**
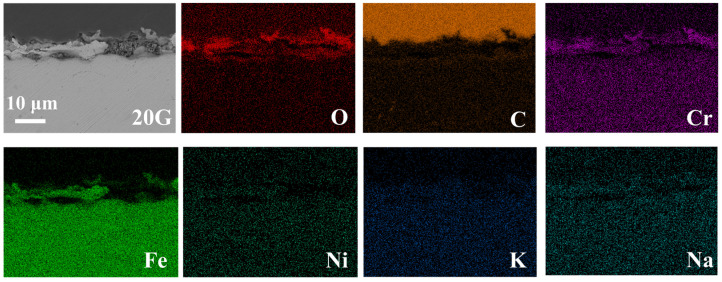
Cross-sectional SEM image and the elemental distribution of 20G alloy after corrosion in molten LiCl-NaCl-KCl at 700 °C for 50 h.

Figure 2 shows the cross-sectional EDS maps of the 20G after corrosion at 700 °C in the LiCl-NaCl-KCl melt. It is seen that 20G exhibits significant corrosion in the eutectic LiCl-NaCl-KCl melt at 700 °C. A loose corrosion oxide layer is formed on the surface of 20G. Based on the element distributions in Figure 2, the major components of the corrosion products are rich in Fe, Cr and O, suggesting that the corrosion oxides should mainly be composed of Fe-rich oxide and/or Cr-rich oxide. This is due to the fact that the corrosion experiment was not conducted in an inert gas. The O_2_ and moisture from the atmosphere are inevitable to react with 20G to form oxides in the molten salt system. However, the presence of Cl^−^ in molten salt could react with oxides and form metal chlorides, causing the oxide films to crack and become loose and porous and ultimately ineffective in providing protection to the steel. As the corrosion progresses, a new substrate is continuously exposed to the salt and corroded, resulting in continuous dissolution of the 20G.

**Figure 3:**
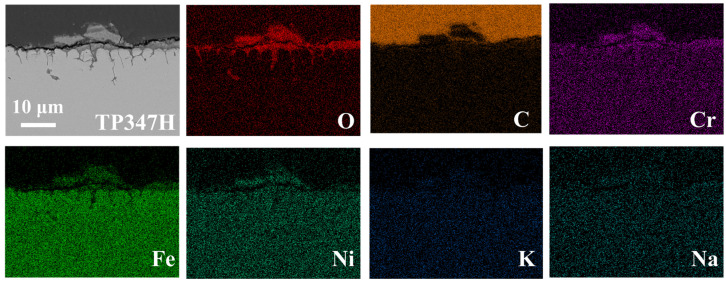
Cross-sectional SEM image and the elemental distribution of TP347H alloy after corrosion in molten LiCl-NaCl-KCl at 700 °C for 50 h.

Figure 3 shows the EDS analysis results of TP347H steel after 50 h corrosion in LiCl-NaCl-KCl melt. A non-protective outer oxide layer is observed in the image, with a notably higher O content on the surface and at the GBs of the materials. As shown in Figure 3, the corrosion products are enriched in Fe, Cr, Ni and O, indicating the oxidation of Fe, Cr and Ni. As expected, TP347H is a typical high Cr austenitic steel, due to the fact that the corrosion experiment was conducted open to air, and contact with the atmosphere inevitably introduced O_2_ and water vapor into the molten salt environment. The Fe, Ni and Cr readily react with O_2_ or water to form oxides. The oxides will react with chloride and dissolve into the salt, causing the oxide films to crack and be porous. Due to the higher energy state of the GB, it usually acts as the preferred path of element migration. As the corrosion progresses, the oxygen in the chloride salt will diffuse along the grain boundaries into the matrix and form oxides at the GBs.

### 3.2. TEM Analysis of TP347H 

**Figure 4:**
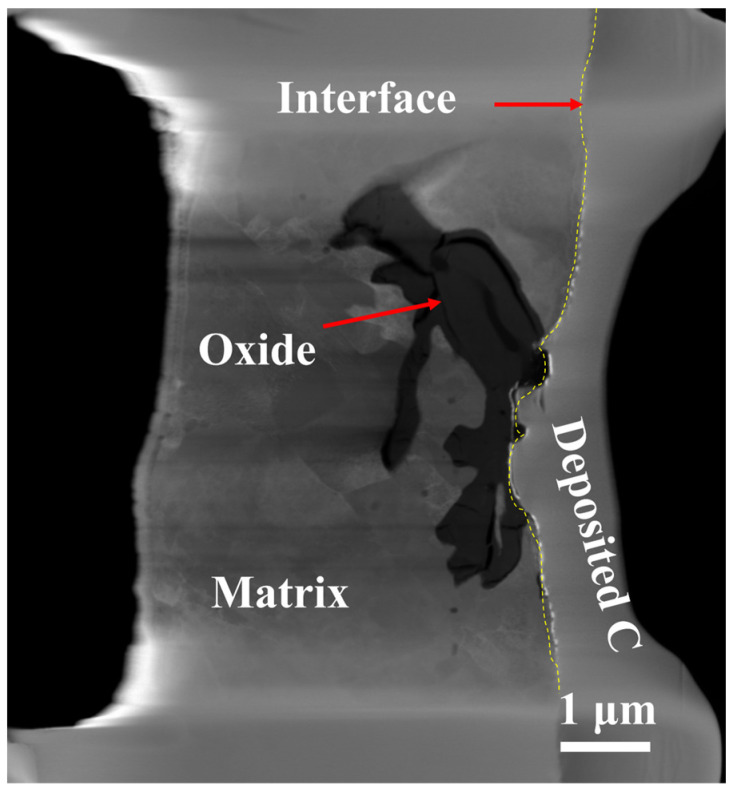
The GB FIB samples of TP347H after corrosion in LiCl-NaCl-KCl ternary eutectic molten chloride salt at 700 °C for 50 h.

The TP347H exhibits significant intergranular cracking, with EDS showing a notable absence of Fe elements at the GBs. This absence can be attributed to the initial adsorption of oxygen on the metal surface, leading to the formation of an outer oxide layer associated with the outward diffusion of iron. Simultaneously, oxygen in the chloride salt will diffuse along the GBs into the matrix and form nano-sized oxides at the GBs. To further illustrate the configuration and formation of oxides within the GBs, a typical oxide formed at the GB of the TP347H was lifted out using FIB technology and characterized using TEM, as shown in Figure 4. In the image, the steel substrate is shown in gray, and the black regions clearly represent the oxides at the GBs.

**Figure 5:**
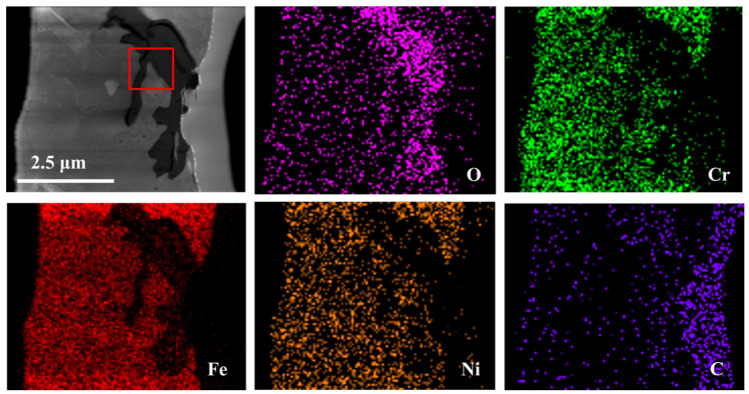
TEM image and corresponding EDS mappings of TP347H after corrosion in LiCl-NaCl-KCl ternary eutectic molten chloride salt at 700 °C for 50 h (Note that the enriched C on the right side of the image originates from the deposited C used to prepare TEM sample).

**Figure 6:**
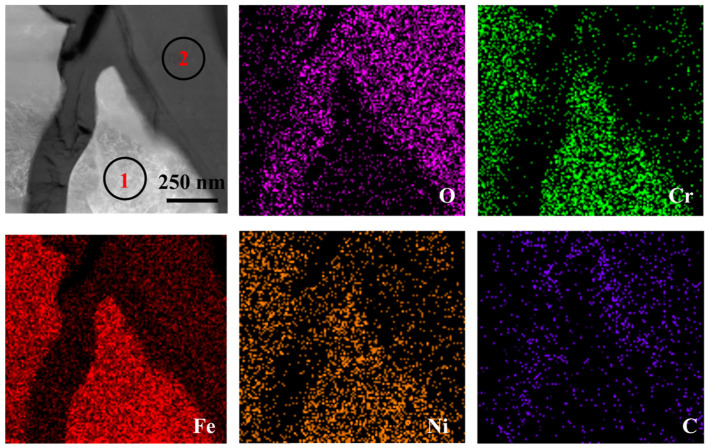
Zoom-in analysis of the red square in Figure 5 and corresponding GB element distribution of TP347H after corrosion in LiCl-NaCl-KCl ternary eutectic molten chloride salt at 700 °C for 50 h. Note that the circles 1 and 2 are the EDS point analysis positions in Table 2.

Figure 5 presents the TEM image and corresponding EDS mappings of TP347H after corrosion in LiCl-NaCl-KCl salt at 700 °C for 50 h. It is observed that the dark region at the GB is enriched in O and with small amounts of Fe and Cr, suggesting the formation of oxides during the corrosion experiment. To confirm the presence of GB oxides, a zoon-in analysis of the red square in Figure 5 was conducted. As shown in Figure 6, the precipitate at the GB consists of O, Cr, Ni and Fe. Further EDS point analysis reveals that the chemical composition of the dark region at the GB contains 32.6 wt.% O, 13.8 wt.% Cr, 37.9 wt.% Fe and 10.8 wt.% Ni, as shown in Table 2. This composition suggests that the oxide could be Fe-Cr-Ni spinel, which is the most common oxide according to the analysis of references [33,34,35]. Additionally, there is C enrichment on the GB of TP347H (Figure 6), possibly related to the formation of Cr-carbides. It is known that the high temperature of 700 °C causes carbon atoms to diffuse to the GBs at austenitic stainless steel, forming high-carbide compounds and accumulating at the GBs [36]. 

**Figure 7:**
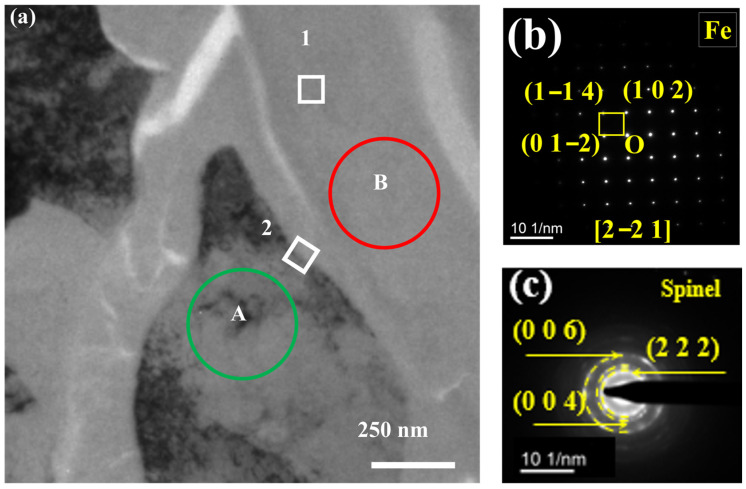
(**a**) TEM images of post-experiment TP347H and (**b**,**c**) selected area electron diffraction patterns analysis of regions of circles A and B in (**a**), respectively.

To accurately determine the structure and composition of the oxide, the precipitates formed at the GB of the TP347H in Figure 7a were further examined using TEM. The substrate phase of region A in Figure 7a was determined by selected area electron diffraction (SAED), and the result reveals that the matrix is a typical Fe FCC phase (Figure 7b). As shown in Figure 7c, the poly-crystalline ring indicates that the precipitate oxide at the GB is mainly polycrystalline with a spinel structure.

**Figure 8:**
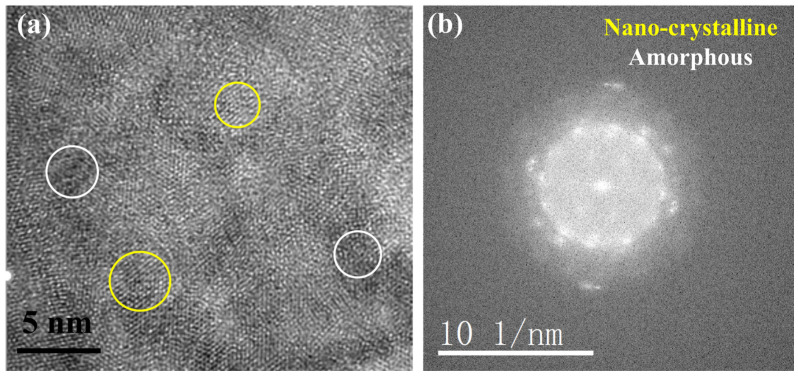
(**a**) The HRTEM image corresponding to the white square 1 in Figure 7a and (**b**) the FFT pattern images. Nano-crystalline area is indicated by yellow circles and amorphous area is indicated by white circles.

**Figure 9:**
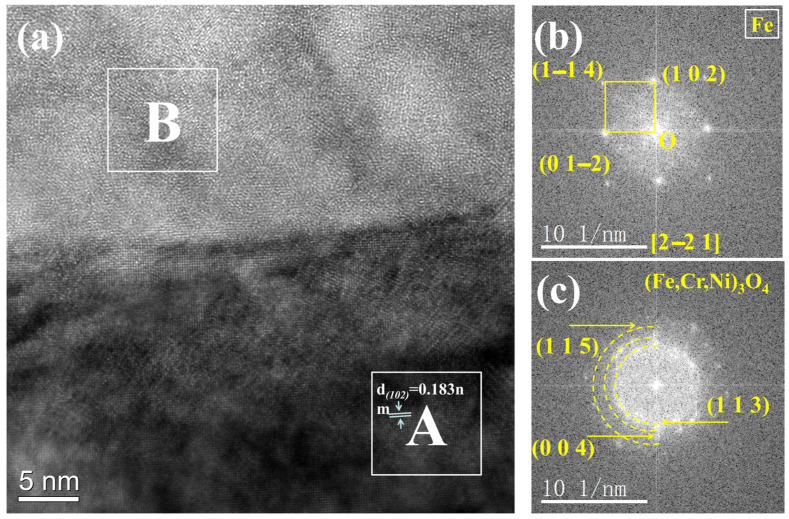
(**a**) The HRTEM image corresponding to the white square 2 in Figure 7a and (**b**,**c**) the FFT pattern analysis of regions A and B in (**a**).

To further determine the structure of the oxide, a high-resolution analysis of region 1 in Figure 7a was conducted, and the results are shown in Figure 8. It is evident that the oxide formed at the GB is nano-sized polycrystalline. Fourier transformed (FFT) revealed that the oxides at the GBs were polycrystalline mixed with amorphous structure, as shown in Figure 8. Figure 9 displays the high-resolution analysis of the oxide/matrix interface (region 2 in Figure 7a), FFT patter images indicate that the TP347H matrix is arranged fairly well. In Figure 9b, the crystal face spacing measured in some regions is determined to be 0.183 nm, indicative of the Fe FCC phase. For the oxide area B in Figure 9a, FFT analysis reveals that oxides are polycrystals. Several distinct orientations were found in the polycrystalline ring. The calibration results showed that oxide is consistent with the structure of spinel. Combined with the EDS results in Table 2, it confirms that the oxide formed at the GB of TP347H after exposure to high-temperature molten chloride salt is (Fe_3-x-y_Cr_x_Ni_y_)O_4_ spinel [33].

## 4. Discussion

In this study, the corrosion behavior of 20G and TP347H alloys in molten chloride salt was investigated. The corrosion rates of these materials in molten chloride salt are high and are affected by the formation of surface oxides on the alloys. The 20G exhibits uniform surface corrosion with a maximum corrosion depth of approximately 13.4 µm (Figure 1). In the austenitic steel TP347H, the higher Cr content promotes the formation of Cr-oxide on its surface, providing better corrosion resistance compared to 20G carbon steel. However, due to the highly corrosive nature of chloride salts, the Cl^−^ in the chloride salt can react with oxides and form metal chlorides, causing the oxide films to crack and become loose and porous, thereby losing their protection to the steel (Figure 1 and Figure 3). As the corrosion progresses, the new substrate is continuously exposed to the salt and corroded. The main corrosion mechanism for carbon steel and austenitic steel is as follows.

During the initial stage of exposure in high-temperature molten chloride salt environments, an oxide film formed on the surface of iron-based materials. As the corrosion experiment was conducted open to the air, O_2_ is readily dissolved into the salt and reacts with the metal to form oxides through the following reaction [37,38].

xMe + y/2O_2_ = Me_x_O_y_ (1)

(Me = Cr, Fe, Ni, etc.). The oxide film formed on the surface of iron-based materials does indeed provide a certain level of protection, slowing down the corrosion rate of the material.

However, the alloying elements within the oxide cannot be stabilized film in the high-temperature chloride molten salt. They can react with Cl^−^ and dissolve into the molten salt, as described by the following reaction [38,39]:

Me_x_O_y_ + 2xCl^−^ = xMeCl_2_ + yO^2−^ (2)

This reaction causes the oxide films to crack, become loose, and porous, thereby diminishing their ability to provide effective protection, as illustrated in Figure 1.

As the oxide film on the Fe-based steel surface dissolved and deteriorated, the chloride molten salt infiltrated into the film and came into direct contact with the matrix. Moisture is the most common impurity in molten salt, particularly in salts like KCl that has a strong water absorption property and it is very difficult to completely remove the water from molten salt [40]. In a high-temperature chloride salt environment, moisture and chloride salt can react to form HCl through the following reaction [38,40]:

MCl(l) + H_2_O(g) = MOH(l) + HCl(g) (3)

where (M = Li, Na, K), the HCl will be partially dissolved into the molten salt and further induces the dissolution of the alloy [38]. As the corrosion progresses, the new substrate is continuously exposed to the salt, resulting in ongoing corrosion and subsequent dissolution of the alloy. This continuous exposure and corrosion process perpetuate the degradation of the material.

In the case of 20G (i.e., low Cr steel), the primary oxide film is Fe-based oxide due to the low Cr content of carbon steel. In molten chloride salts, the Fe-oxide may dissolve into the salt by forming soluble iron chlorides [39]. This dissolution of alloys in the near-surface area creates pathways for the ingress of dissolved oxygen and electrolytes into the substrate, continually deteriorating the steel substrate and causing general corrosion.

Unlike 20G, in the case of TP347H austenitic steel, the higher Cr content of TP347H alloy promotes the formation of Cr-containing spinel oxide and provides better corrosion resistance than 20G carbon steel. Indeed, the protective nature of oxide structures with a high Cr content is higher than that of Fe-oxide [41]. This superiority arises from the unique diffusion characteristics within spinel structures. In spinel, the diffusion of Fe ions occurs through octahedral vacancies. When the concentration of Cr ions is high, Cr preferentially replaces Fe in the octahedral vacancies, substantially reducing the diffusion rate of Fe ions. This reduction occurs because, unlike Fe which can undergo oxidation from 2(II) to 3(III), Cr does not change its oxidation state and could reduce the available diffusion paths for Fe ions [41]. Thereby, the presence of Cr in spinel oxide significantly inhibits the diffusion of Fe elements in both GBs and crystals, leading to a slowdown in the corrosion rate of Fe-based steel.

The formation of high-Cr spinel oxide in austenitic stainless steel is considered the primary reason for its better corrosion resistance than that of carbon steel. However, the Cl^−^ in molten salt reacts with Cr spinel oxides, causing the oxide film to crack and become loose and porous, thereby reducing its effective protection. With the dissolution of oxide film, numerous defects are created, providing fast channels for element diffusion. As the steel continues to be exposed to molten salt, the new substrate is continuously corroded. Due to the higher energy state of the GBs, they usually act as the preferred path of element migration. As the corrosion progresses, oxygen in the chloride salt diffuses along the gran boundaries into the matrix and forms spinel by the following reaction at the GBs [32,33].

2O_2_ + (3−x−y)Fe + xCr + yNi = (Fe_3−x−y_Cr_x_Ni_y_)O_4_ (4)

Due to the different corrosion potential between oxide and its surrounding matrix, micro-galvanic corrosion can occur, accelerating the intergranular corrosion of austenitic alloys (Figure 3).

## 5. Conclusions

The corrosion behavior of 20G and TP347H materials was investigated in molten LiCl-NaCl-KCl salt at 700 °C. The results show that the corrosion rates of these materials in molten chloride salt are high and are strongly affected by the alloying surface oxide formation. The main findings are as follows:

(1) 20G shows uniform surface corrosion with almost no protective oxide formation on the surface, the corrosion depth reaches 13.4 µm after exposure in molten chloride salt at 700 °C for 50 h.
(2) Unlike 20G, in the case of TP347H austenitic steel, the higher Cr content of TP347H alloy promotes the formation of Cr-containing spinel oxide and provides better corrosion resistance than 20G carbon steel.
(3) Due to the highly corrosive nature of molten chloride salts, the Cl^−^ in molten salt could react with oxide, causing the oxide films to crack and become loose and porous, thereby reducing its effective protection for Fe-based alloys.
(4) Due to the higher energy state of the GB, it usually acts as the preferred path of element migration. After the oxide film diminishes their protective ability, the oxygen impurity in the chloride salt can diffuse along the GBs into the TP347H matrix and form (Fe_3-x-y_Cr_x_Ni_y_)O_4_ spinel at the GBs, accelerating the oxidation and intergranular corrosion of austenitic steel in molten chloride salts.

## Figures and Tables

**Table 1:** The compositions (wt.%) of 20G and 347H used in this study.

	C	Si	Mn	S	P	Cr	Ni	Other
20G	0.19	0.20	0.35	0.014	0.012	0.10	0.04	Mo:0.08
TP347H	0.04~0.1	≤0.75	≤2.00	≤0.03	≤0.035	18~20	10~13	Nb:0.8

**Table 2:** Chemical composition (wt.%) of the positions in Figure 6.

Position	O	Cr	Fe	Ni	C
1	32.6	13.8	37.9	10.8	4.9
2	0.5	10.9	69.1	14.8	4.7

## Data Availability

The data that support the findings of this study are available from the corresponding authors upon request.
